# The effects of communication training on communication competence – a 360° evaluation

**DOI:** 10.1186/s12909-025-07676-9

**Published:** 2025-07-31

**Authors:** Lisa Zöll, James Deschner, Anke Hollinderbäumer, Thomas Nowak, Tamara Zajontz, Lina Behling, Sabine Fischbeck, Uwe Schmidt, Thekla J. Pfeiffer-Grötz

**Affiliations:** 1 Department of Periodontology and Operative Dentistry, University Medical Center, Johannes Gutenberg University, 55131 Mainz, Germany; 2 University Medical Center, University of Mainz, Rudolf Frey Lernklinik, 55131 Mainz, Germany; 3 Center for Quality Assurance and Development (ZQ), Johannes Gutenberg University Mainz, 55124 Mainz, Germany; 4 Department of Psychosomatic Medicine and Psychotherapy, Medical Psychology and Medical Sociology, University Medical Center, Johannes Gutenberg- University, 55099 Mainz, Germany

**Keywords:** Communication skills, Dental curriculum, Dental education, 360° evaluation

## Abstract

Communication skills training is essential in dental education, but training effects are often difficult to detect. The aim of this study was to assess the effect of communication training through 360° evaluation. To this end, 127 dental students were questioned with their treatment partners, their course assistants and their patients. 35 male and 92 female students took part in the study from April 2021 to February 2022.

The students’ self-assessment of their communication skills was carried out using a questionnaire at two different points in time. In addition, external assessments of communication skills were obtained from treatment partners, course tutors and patients at one point in time. A five-point Likert scale was available to all participants.

Our study shows that communication training in dental curriculum improves the students’ self-assessment of their communication skills. In addition, some effects were also measurable in the external assessment by the treatment partners and the course tutors. The patient survey showed very good results in both groups.

In summary a one-time communication training course significantly improves students’ self-perception of their communication skills, especially when it comes to more complex skills such as conversation structure and nonverbal communication. Treatment partners and course tutors also confirm improvements, while patients were unable to perceive any differences. The study shows that simple skills can be improved even without training, but complex skills require targeted training. However, longer training and further research are needed for sustainable behavioral changes and objective assessments.

**Supplementary Information:**

The online version contains supplementary material available at 10.1186/s12909-025-07676-9.

## Introduction

Communication with regard to a good dentist-patient relationship is an important factor in dental treatment. If this is a good relationship between dentist and patient, it serves to improve the patient’s sense of security and control. It also reduces the patient’s perceived stress and anxiety during treatment [1]. Appropriate communication offers enormous advantages for the patient, creating an improved basis of trust with verbal and non-verbal styles. Good communication between dentist and patient also has a significant influence on the treatment process and the success of the treatment [2, 3]. The limited ability to communicate during treatment is an important aspect of dental treatment. Structured communication is very important for both the dentist and the patient. It is therefore necessary to have certain communication skills and abilities as a dentist and to be taught these during dental training and to train their practical application. This study examines the benefits of a 360° evaluation of communication training in dental education. In this assessment, the various aspects of communication competence after such communication training are evaluated not only by the student themselves, but also by the course tutor, the treatment partner and the patient. Such an evaluation from different perspectives should provide a more objective assessment of the potential of communication training. This study uses the following data from the end of the last study, which was published under the following title “Effect of a One-Time Communication Training Session on Dental Studentsʼ Self-Efficacy Expectancy” [4].

## Materials and methods

### Sample composition

For the study, students in the first and last clinical semester, i.e. the 7th and 10th semesters, of the dental curriculum at Johannes Gutenberg University Mainz were surveyed from April 2021 to July 2021 and October 2021 to February 2022. In both semesters, the students were divided into an intervention group and a control group (CG). A total of 127 out of 159 students participated in the study. Twenty-nine students had to be excluded due to incomplete data. The IG included 64 students, while the CG included 63 students. Furthermore, the course tutors, the treatment partners and the treated patients were interviewed for this study as a 360° evaluation during the period mentioned. The study was previously presented to the ethics committee of the State Medical Association of Rhineland-Palatinate. All participants were fully informed and participated voluntarily in the study.

### Intervention and control groups

An online theory module and one unit of face-to-face teaching were carried out. Self-assessed learning success was evaluated by means of questionnaires completed by the students before and after the module [4].

At the beginning of each semester, the students in both groups received a questionnaire for self-assessment of their communication skills, which was completed pseudonymously (time T0). A lecture was then made available in the existing online learning platform for self-study (theory module). In the third week of the semester, the practical unit of communicative face-to-face teaching took place for the intervention group. In this practical module, the students conducted counseling sessions with drama patients who portrayed different characters. In the fifth and sixth week of the semester, the self-assessment questionnaire on communicative skills was again distributed to both groups as a pseudonymized questionnaire (time T1). This made it possible to compare the self-assessment from T0 to T1 in both groups. The control group did not receive any interventions or information between T0 and T1. Only after T1 were they provided with the theory module and also received a practical session with drama patients [4].

### Study participants 360° evaluation

Throughout the entire period, the course tutor and the treatment partner were surveyed at the end of each semester as part of a feedback questionnaire. In the middle of the semester, after the practice unit for the intervention group had taken place, all patients were also surveyed over a period of 4 weeks as part of a feedback questionnaire. All questionnaires were posted anonymously in a mailbox provided for this purpose. It was therefore not possible to trace the respondent.

### Online theory module

The online theory module provided technical background on communication techniques in the form of a PowerPoint presentation given by two dentists (T.J.G., L.Z.). The online theory module lasted twice 45 min. This online module was based on the following learning objectives of the National Competence-Based Learning Objectives Catalog for Dentistry (NKLZ), which describes the desired profile of dental graduates in Germany:

- Students can establish and maintain a positive, sustainable and trusting dentist-patient relationship through their communicative actions [3].
- Students can structure patient conversations from start to finish. They can weight the parts differently depending on the type of conversation [3].
- Students know the importance of non-verbal communication and use positive, non-verbal signals [3].
- Students are able to use suitable conversation and questioning techniques as appropriate to the situation [3].
- Students are able to deal appropriately with emotionally challenging situations in the dentist-patient relationship [3].

The lecture was developed in cooperation with the Center for Quality Assurance and Development and the Department of Medical Psychology and Medical Sociology at the Mainz University Medical Center [4].

### Practical teaching unit

In the practical training of the intervention group, the students were confronted with a standardized training situation. The practical module was developed together with the Rudolf Frey Learning Clinic. Actors were available who had already worked with similar educational activities as part of the communication module in the human medicine course. All actors were given the same starting situation and each assigned a role in the form of a character trait. The starting situation for each role was a patient who had already presented at the outpatient clinic and had been diagnosed with periodontitis. The conversation to be conducted was a consultation and explanation of periodontitis treatment. All of the people portrayed had type 2 diabetes as their underlying disease. The actors portrayed the following people:

- anxious patient.
- angry patient after a long wait.
- patient over-informed by the Internet.
- patient with possible HIV infection.
- a frequent talker.
- quiet patient who is difficult to talk to.

These figures offered the opportunity to simulate emerging conflict situations. Each student had the opportunity to work on at least one case study. The students then received feedback from the patients involved, the assistant doctor and their fellow students. Each figure was recorded on video as a case study with the consent of the respective student [4].

The control group received the practice unit in the same form after completion of the survey at time T1.

At the end of the semester, the students again received the self-assessment questionnaire, which could be assigned to the previously completed questionnaire by pseudonymization.

All questionnaires were developed for this study in collaboration with the Center for Quality Assurance and Development. The self-assessment questionnaire had previously been used and validated in the study “Effect of a One-Time Communication Training Session on Dental Studentsʼ Self-Efficacy Expectancy” [4]. The questionnaires for external evaluation by treatment partners, course tutors and patients were derived from the self-assessment questionnaire and also validated. Every questionnaire was then transferred to survey software (EvaSys, Electric Paper Evaluationssysteme GmbH, Lüneburg, Germany). This allowed the questionnaires to be evaluated electronically. In order to pseudonymize the questionnaires and then reassemble them anonymously, a personal code was created for each student. This code consisted of the date of birth and the names of the parents and the student and was entered on the respective questionnaire. The code was only known to the student. The questionnaire recorded socio-demographic data and the students’ self-assessment of their communication skills [4].

### Sociodemographic parameters

The following socio-demographic parameters were collected: Age, gender and mother tongue.

#### Self-assessment of communicative competence

Ten statements based on the learning objectives were formulated to assess the students’ own communication skills (supplementary file 1).

### 360° evaluation

#### External assessment of communicative competences by the treatment partner

Ten statements with the same content as for the students themselves were formulated to assess the communication skills of the students by the treatment partner (supplementary file 2).

#### External assessment of communicative competences by the course tutor

Ten statements with the same content as for the students themselves were formulated to assess the communication skills of the students by the course tutor (supplementary file 3).

#### External assessment of communicative competences by the patient

Six of the ten statements with the same content as for the students themselves were used to assess the communication skills of the students by the patient (supplementary file 4).

### Statistical analysis

The statistical analysis and presentation of the data was carried out using SPSS Statistics 23 (IBM, Armonk, New York, USA) and Excel Version 2102 (Microsoft, Redmond, Washington, USA) and was supervised by the Institute of Medical Biometry, Epidemiology and Informatics (IMBEI) at the Mainz University Medical Center. A descriptive analysis of the socio-demographic data was carried out. The students’ self-assessment was collected at T0 and T1 in order to record the change in attitude. The mean value comparisons of the intervention group and the control group of the students were analyzed using Wilcoxon signed-rank test for paired samples, as a normal distribution could not be assumed. For the statistical analysis of the 360° evaluation of the students, treatment partners, the course tutors and the patients, the data of the intervention group and the control group were compared with each other at time T1 using a Mann-Whitney U test. The significance level was set at *p* < 0.05 in each case.

## Results

### Sociodemografic parameters

One hundred and twenty-seven students participated in the study of whom 35 (27.60%) were male and 92 (72.40%) female. After the exclusion of 29 people due to incomplete data, 81.41% of all participants were included. The average age was 24.68 years. Ninety-one students stated German as their mother tongue (Table 1).

**Table 1:** socio-demographic parameters of the students

feature	characteristic	number (*N*)
age	years	24.68 years (SD ± 3.249)
gender	male	35
	female	92
	divers	0
mother tongue	German	91
	other	23
	not specified	13

### Self-assessment of communicative competences

**Fig. 1:**
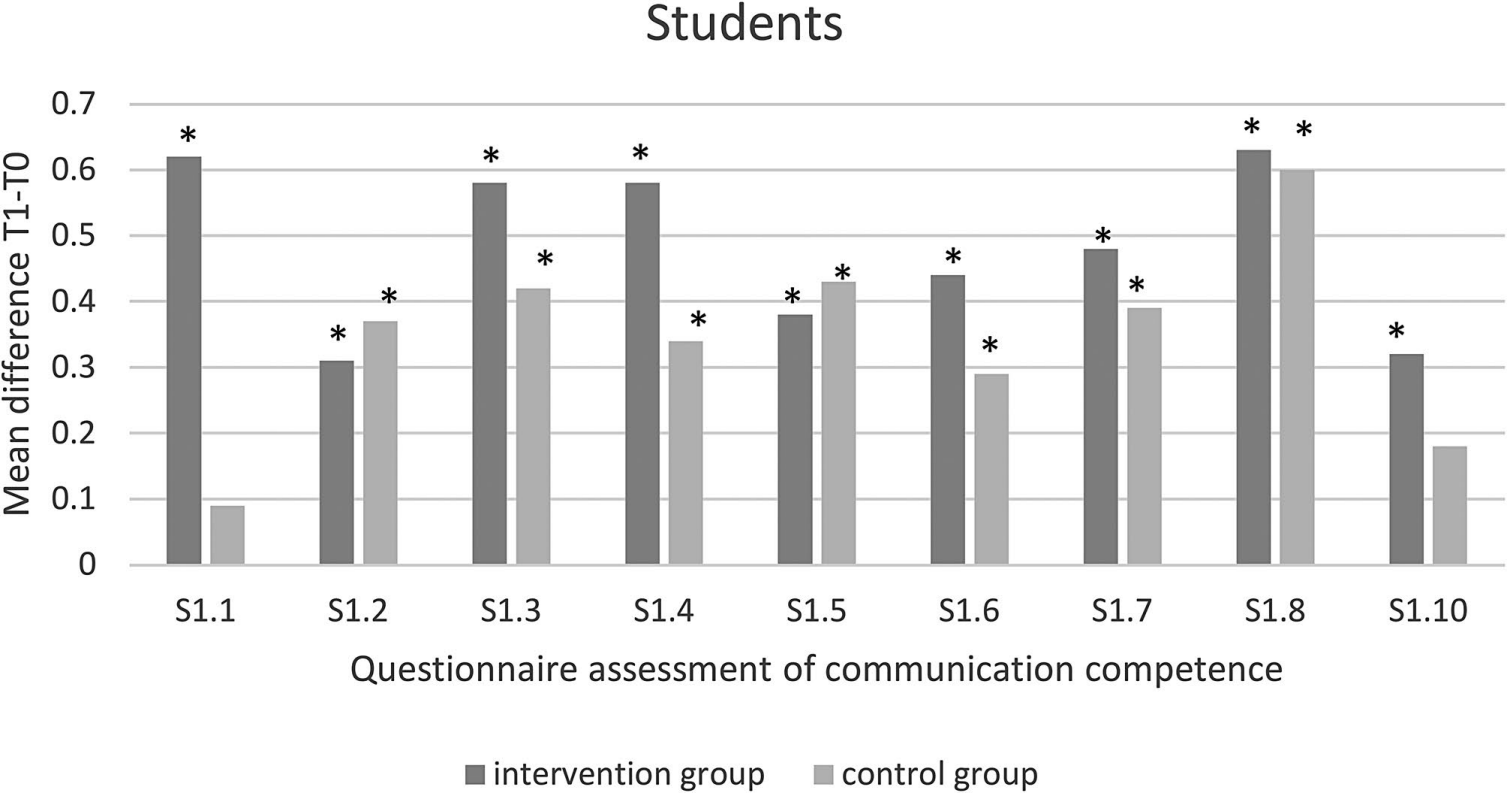
Questionnaire assessment of communicative competence- students (*significant at *p* < 0.05)

Our results show that all parameters in the intervention group (communication training) improved significantly between survey time points T0 and T1 (S1.1: *p* < 0.001; S1.2: *p* = 0.005; S1.3: *p* < 0.001; S1.4: *p* < 0.001; S1.5: *p* = 0.004; S1.6: *p* = 0.002; S1.7: *p* < 0.001; S1.8: *p* < 0.001; S1.10: *p* = 0.009) (Fig. 1).

The control group showed significant improvements in the students’ self-assessments of questions 1.2–1.8. (S1.2: *p* = 0.004; S1.3: *p* = 0.011; S1.4: *p* = 0.042; S1.5: *p* = 0.021; S1.6 *p* = 0.023; S1.7: *p* < 0.001; S1.8: *p* = 0.012) between both time points.

An intergroup comparison of the results of the students’ self-assessments was carried out between the intervention and control group at the survey time point T1. The statistical comparison revealed no statistically significant differences between the two groups at this one point in time. When comparing the changes in attitude between time points T0 and T1, significant changes were found within both groups, which were compared descriptively.

### External assessment of communicative competences by the treatment partner

**Fig. 2:**
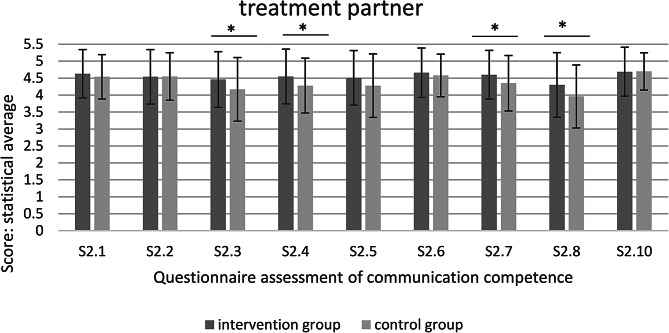
Questionnaire assessment of communication competence– treatment partner (* significant at *p* < 0.05)

In the comparison between intervention and control group, significant improvements of the intervention group are shown in the individual questions 2.3 (*p* = 0.046), 2.4 (*p* = 0.015), 2.7 (*p* = 0.034) and 2.8 (*p* = 0.009) (Fig. 2).

### External assessment of communicative competences by the course tutor

**Fig. 3:**
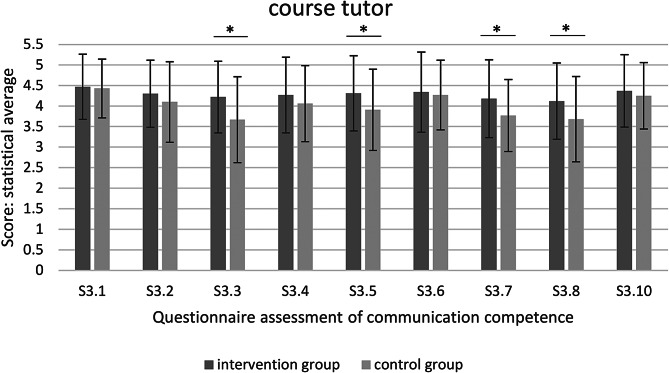
Questionnaire assessment of communication competence– course tutor (* significant at *p* < 0.05)

The intervention group was rated significantly better by the course assistants in the following parameters compared to the control group: 3.3 (*p* < 0.001), 3.5 (*p* = 0.007), 3.7 (*p* = 0.001) and 3.8. (*p* = 0.006) (Fig. 3).

### External assessment of communicative competences by the patient

**Fig. 4:**
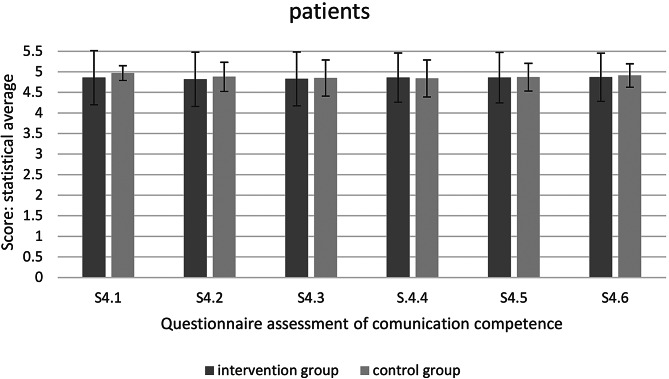
Questionnaire assessment of communication competence– patients (* significant at *p* < 0.05)

The interview with the patients did not reveal any statistically significant differences between the control and intervention groups. None of the individual questions showed statistically significant differences between both groups (Fig. 4).

## Discussion

The students in the intervention group all rated their own communication skills as significantly better after the communication training than at the start of the semester. In contrast, the control group also rated many skills as significantly better. There are also clear differences in the assessment of the amount of skill growth between the two groups.

The survey of the treatment partner shows that individual skills (S2.3, S2.4, S2.7, S2.8) are rated significantly better than in the control group.

Looking at the results of the course tutors, the intervention group is rated significantly better in four parameters (S3.3, S3.5, S3.7, S3.8) than the control group.

There were no significant differences between the control and intervention groups in the patient survey. In addition, all evaluations are very positive.

A stage model can be derived from the results of our study. The stage model is based exclusively from available results in this study and classifies the communicative learning objectives according to their complexity. Three different competence levels are defined: The lowest level is formed by learning objectives that contain simple instructions for action. These are easy to understand and can be learned without training. At the middle level are skills that are more complex and require precise instructions. These skills can be improved through targeted training. The most complex level is made up of learning objectives that can only be learned through training. These require a deeper understanding, guided exercises and feedback.

Our study shows that students rate their own communication skills significantly better after a one-time communication training course than at the beginning of the semester. In comparison, the control group without training also evaluated questions 1.2–1.8 as statistically significantly better. The control group even rated their increase in competence in questions 1.2 and 1.5 higher than the intervention group.

Questions 1.2 and 1.5 include involving patients in decisions and summarizing information for patients, which form the lowest level. These are learning objectives that contain concrete instructions for action and are therefore easier to understand. Due to these concrete instructions and the fact that the students from the control group were informed about their participation in the study, a Hawthorne effect can be assumed [5].

Questions 1.3, 1.4, 1.6, 1.7 and 1.8 were rated significantly better by both groups at time T1 than at time T0. However, the intervention group rated the increase in competence in conversation structure, the use of positive non-verbal signals, responding to patients’ emotions, dealing with different types of behavior and the use of questioning techniques higher than the control group. These learning objectives contain clear instructions and are therefore rated as better by all students. However, the application is more complex and the training specifically trains and improves these skills. For this reason, the intervention group achieved a higher increase in competence. These skills represent the medium complexity level.

The highest level of complexity comprises questions 1.1 and 1.10, which were only rated as significantly better in the intervention group. Both questions address complex skills such as relationship building and the ability of patients to describe their concerns themselves. The results clearly show that these skills can only be learned through targeted communication training. In particular, building a positive and trusting dentist-patient relationship is the foundation of any dental treatment, as illustrated by the Calgary-Cambridge concept [6]. In addition, the study by Abrahamsson et al. shows that a good relationship between dentist and patient leads to an improvement in the patient’s sense of security and control, as well as reducing perceived stress and anxiety during treatment [1].

Question 1.10 examines the ability of patients to describe their concerns themselves. Langewitz et al. investigated the initial speaking time of patients and found that most patients described their visit request after 92 s. This initial speaking time without interruption is of great importance, as this is when the most important information is conveyed by the patient [7].

A study by Najstöm et al. demonstrates that students are able to assess their own communication skills in a differentiated manner and can therefore take an active role in developing their own skills. The self-assessment methodology is particularly recommended as an educational method [8]. The study by Kruse et al. also shows an improvement in self-assessment of communicative skills due to the increase in treatment experience. However, in discussions with simulation patients, students who had completed communication training performed significantly better than practitioners with many years of professional experience without communication training [9]. The findings of Graf et al. highlight that there are differences between self-perception and external perception of communication skills and that both should therefore be used in combination when teaching communication skills [10]. For this reason, in addition to the students’ self-assessment, external assessments were also used for evaluation.

All dental treatments in the student course are carried out in pairs. The practitioner and treatment partner form a student treatment team and assist each other. The respective treatment partners were always assigned to the same study group. As the treatment partner assists with each treatment, they can observe the practitioner throughout the semester. The external assessment of the treatment partner can be rated as good in both groups. However, the treatment partners rated the individual questions 2.3, 2.4, 2.7 and 2.8 as significantly better in the intervention group. The individual questions relate to the structure of the conversation, the use of positive non-verbal signals, dealing with different types of behavior and the appropriate use of questioning techniques. All of these skills represent the medium complexity level, which includes clear instructions. However, significant improvement in these skills can only be achieved through targeted training. Good observability is elementary for external evaluation. The structure of the conversation and the use of questioning techniques are subject to clear models and concepts that are directly and easily observable [6, 11]. In addition, non-verbal signals are easily recognizable and simple to evaluate. Dealing with different characters is the core element of the simulation exercises and was specifically trained by the intervention group.

Overall, all external observations by the treatment partners were very positive and benevolent. The students are free to choose their treatment partners, have often known them since the start of the study and are frequently friends. They are also in the same study group. For this reason, an observation bias can be assumed [12].

A further external assessment was carried out by the course tutors. They are responsible for the patient treatment of 6–7 students at the same time and evaluate individual treatment steps. For this reason, they only ever have selective insights into the treatments of individual students. The external assessment by the course tutors was also very positive for both groups. Compared to the control group, the intervention group was rated as significantly better in individual questions 3.3, 3.5, 3.7 and 3.8. These individual questions cover the topics of conversation structure, summarizing information, dealing with behavior and the use of questioning techniques, which represent the medium complexity level. These skills are easy to observe and can be assessed by asking patients brief questions. The course tutors cannot assess more complex skills, as these require a longer observation period. A course tutor supervises up to seven students at the same time and it is therefore unable to follow all the students´ conversations. For this reason, observation periods are always sequential. In addition, these are end-of-semester assessments in which the course tutors assess all their students. For this reason, the phenomenon of peer assessment can quickly arise, in which all students are assessed similarly. In addition, course tutors have not received any training in skills observation. Observation skills require training and time in order to be able to assess skills in a differentiated manner. In particular, the targeted addressing of observations offers room for possible improvement. Without training, there is otherwise an implicit inference of competencies without having specifically observed them [13].

The informative value of external assessments is heavily dependent on the observational skills of the course tutors and the tutory relationship.

The patient survey revealed no significant differences between the control and intervention groups. All students were rated very well by the patients. The lowest mean score was 4.82 with a maximum score of 5. All patients felt well treated in the student course. A differentiated assessment of communication skills by the patient is not possible. The reasons for this are the lack of expertise, lack of comparison between students, fear of negative assessments and low ability to differentiate. The patients who are treated in the student course are often very patient and benevolent, which explains the very good results. In addition, the survey draws attention to the students’ behavior, which favors a positive assessment.

### Strengths and limitations

The main limitation of the study is that the results are based on the students´ self-assessment. This study does not use objective methods to verify the actual behavioural changes of the students. Furthermore no patient-related effects are measured and no objective evaluation of the patient interviews with a validated measuring instrument was conducted. Looking at the effects of the training according to Kirkpatrick´s evaluation methodology, it becomes apparent that only level two of four has been archieved. The behavioural changes and the desired results are not examined. As a result, the impact of the training remains at the learning level [14].

Students´ self-assessment is influenced by various factors. In particular, social desirability causes students to assess themselves more positively, as they tend to give answers that are likely to make the respondent look good [15]. Answers thar are considered inappropriate in society are therefore not answered truthfully and are consequently systematically over- or underrated [16].

The meta-analysis by Nieminen et al. on student self-assessments clearly indicates that the validity is highly dependent on context, content and design. Structured reflection and feedback can significantly increase the validity of self-assessments and the learning potential [17].

This was the only event on communication skills for students, which may have had a positive influence on the assessment of their own skills. Positive mood during the post-training survey influences the response results. Askim et al. showed that affective state can be a bias in subjective self-assessments [18].

In addition, it was often difficult to assign the pseudonymized codes during the evaluation, which meant that twenty-nine students had to be excluded.

The methodology of 360° evaluation should be critically examined. Eichinger et al. showed that accurate and objective assessment is only possible if you have known the person long enough, otherwise the first impression is overrated [19].

One strength of the study is that a level model could be derived from the results. Since the stage model was derived solely from the results of this study, further research is needed to generalize the findings on the complexity levels of communicative skills. Our study has shown that a one-time communication training course can already lead to a subjective increase in competence, which has a positive effect in many aspects. The students feel more self-confident which is influences their academic performance, the quality of their treatments and the mental health of the students in a positive way [20]. In addition, the 360° evaluation perspective provides valuable feedback from all groups of people involved and can help students better understand how they are perceived by patients, course tutors and colleagues [21]. The review by Liebenow et al. clearly shows that feedback increases the accuracy of self-assessments [22]. Self-assessment and feedback are two effective tools for improving communication skills [8, 23]. It is clear that the differentiation of the individual effects between the various groups of people surveyed is decreasing. The differences in the students’ self-assessments are most clearly visible, followed by the external assessments by the treatment partners. The effects in the external assessments by the course tutors are far less pronounced and the patients were unable to identify any differences.

It would be interesting to compare the subjective assessments with objectifiable parameters and to investigate the effects of a longer intervention in terms of longitudinal communication training.

## Conclusion

In summary, our study shows that a one-time communication training course has a positive effect on students’ self-assessment of their communication skills. Some of these effects are also perceived by the treatment partners and course tutors. However, the patients are unable to differentiate any differences.

The study points out limitations, such as the exclusive use of self-assessment without objective behaviour measurement and possible distortions due to social desirability or observer effects. Nevertheless, it shows that a one-time communication training course strengthens students’ subjective sense of competence and can have positive effects on their self-confidence and treatment success. A combination of self-assessment and external assessment, as well as longer training, could lead to even better results in the future.

## Electronic supplementary material

Below is the link to the electronic supplementary material.

Supplementary Material 1

## Data Availability

The datasets used and analysed during the current study are available from the corresponding author on reasonable request.

## Abbreviations

CG: Control group
EvaSys: Electric paper evaluationsystems
HIV: Human immunodeficiency virus
IG: Intervention group
NKLZ: National competence-based-learining objectives catalogue
ZQ: Center for quality assurance & development
