# Supplementary material for: The effects of communication training on communication competence – a 360° evaluation
Source: BMC Med Educ. 2025 Jul 31;25:1128. doi: 10.1186/s12909-025-07676-9 (PMC12312342; doi:10.1186/s12909-025-07676-9)

# Supplementary file

### Supplementary file 1: Self-assessment of communicative competence

Ten statements based on the learning objectives were formulated to assess the students' own communication skills:

1.1: I am able to build up a positive and trusting relationship with the patient.

1.2: I involve the patient in the decision-making process.

1.3: I structure the patient conversations.

1.4: I use positive signals of non-verbal communication.

1.5: I can summarize information for the patient.

1.6: I respond to the patient's emotions.

1.7: I can deal with different behaviors of the patient.

1.8: I can use questioning techniques adequately.

1.9: I have achieved my treatment goal on today's treatment day.

(The question was deleted because no treatment was carried out due to SARS-CoV-2 pandemic).

1.10: I give the patient the opportunity to describe his or her own concerns.

The questions could be answered on a five-point Likert scale from "strongly disagree" (1) to "strongly agree" (5).

### Supplementary file 2: External assessment of communicative competences by the treatment partner

Ten statements with the same content as for the students themselves were formulated to assess the communication skills of the students by the treatment partner:

2.1: The student is able to build up a positive and trusting relationship with the patient.

2.2: The student involves the patient in the decision-making process.

2.3: The student structures the patient conversations.

2.4: The student uses positive signals of non-verbal communication.

2.5: The student can summarize information for the patient.

2.6: The student responds to the patient's emotions.

2.7: The student can deal with different behaviors of the patient.

2.8: The student can use questioning techniques adequately.

2.9: The student has achieved my treatment goal on today's treatment day.

(The question was deleted because no treatment was carried out due to SARS-CoV-2 pandemic).

2.10: The student gives the patient the opportunity to describe his or her own concerns.

The questions could be answered by the treatment partner on a five-point Likert scale from "strongly disagree" (1) to "strongly agree" (5).

### Supplementary file 3: External assessment of communicative competences by the course tutor

Ten statements with the same content as for the students themselves were formulated to assess the communication skills of the students by the course tutor:

3.1: The student is able to build up a positive and trusting relationship with the patient.

3.2: The student involves the patient in the decision-making process.

3.3: The student structures the patient conversations.

3.4: The student uses positive signals of non-verbal communication.

3.5: The student can summarize information for the patient.

3.6: The student responds to the patient's emotions.

3.7: The student can deal with different behaviors of the patient.

3.8: The student can use questioning techniques adequately.

3.9: The student has achieved my treatment goal on today's treatment day.

(The question was deleted because no treatment was carried out due to SARS-CoV-2 pandemic).

3.10: The student gives the patient the opportunity to describe his or her own concerns.

The questions could be answered by the course tutor on a five-point Likert scale from "strongly disagree" (1) to "strongly agree" (5).

### Supplementary file 4: External assessment of communicative competences by the patient

Six of the ten statements with the same content as for the students themselves were used to assess the communication skills of the students by the patient:

4.1: The student is able to build up a positive and trusting relationship with me.

4.2: The student involves me in the decision-making process.

4.3: The student structures the conversations.

4.4: The student uses positive signals of non-verbal communication.

4.5: The student can summarize information for me.

4.6: The student responds to my emotions.

The questions could be answered by the patient on a five-point Likert scale from "strongly disagree" (1) to "strongly agree" (5).

Supplementary file

Supplementary file 5: E-mail of the Ethics Committee of the State Medical Association of Rhineland-Palatinate


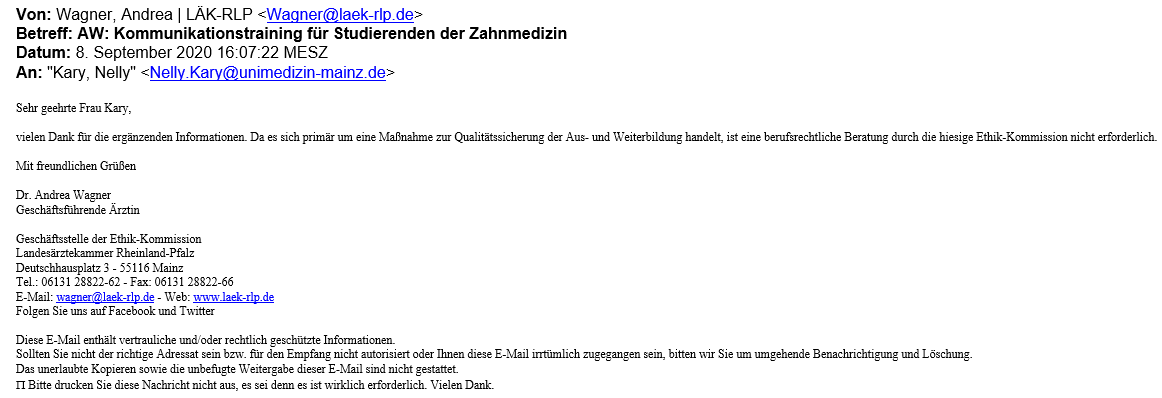

Supplement: Supplementary file 1 — Supplementary Material 1 [file 12909_2025_7676_MOESM1_ESM.docx]
